# Notes and Comments

**Published:** 1893-05

**Authors:** 


### Notes and Comments.[1]

NITROUS-OXIDE-GAS MORTALITY.—Dr. J. D. Thomas, in writing upon the subject of nitrous oxide, says, "Professor Wilbur F. Litch, in an article upon anæsthesia, published in the 'American System of Dentistry,' has made a very careful and complete compilation of all the fatal cases attributed to nitrous oxide, and he there shows eleven cases of death; but in a recapitulation he eliminates four of them from the list as being in no way connected with the gas. Of the seven remaining, you will notice five of them occurred in Europe and but two in this country. If, as they say in stock speculation, we strike an average by dividing twenty-eight, the number of years in which nitrous oxide has been in use, by two, which would give fourteen, and multiply the annual number—750,000—by it, we would have 10,500,000 people who had taken the gas in this country, with but two deaths resulting therefrom. Of these, the first occurred in 1864,—within a year after the beginning of its use. It is said the patient recovered from the effects of the gas and walked into an adjoining room apparently well; he shortly returned and complained of shortness of breath, sank upon a sofa, and expired in a few moments. The coroner's verdict was that death was caused by congestion of the lungs, induced by the inhalation of nitrous oxide.

"It is difficult to comprehend how the inhalation could have produced congestion of the lungs, as an after-effect to have caused immediate death; and I would be inclined to think it more from nervous depression and final heart-failure, which could have been from reaction rather than congestion."

---

NITRATE OF SILVER.—There is much accumulating evidence in regard to the uses of nitrate of silver in dental practice. In addition to its value in the treatment of dental caries, abrasion, etc., it is proving to be quite efficient in the treatment of erosion. Professor Peirce's recent communication to this journal in regard to

---

[1] The assistant editor solicits contributions for this department,—new methods, new remedies and formulas, or any short practical note which may prove of value to the practitioner or student. Address 1506 Arch Street, Philadelphia.

the preparation of nitrate of silver in the form of small pads—by saturating a piece of blotting-pad with a forty-per-cent. solution—is a valuable and timely contribution to the subject. He neglected, however, to speak of the necessity of protecting it from the atmosphere and sunlight, as it is decomposed by the influence of these. It should therefore be kept in a dark-colored bottle having a ground-glass stopper.

---

The Genese Crown.—Dr. Genese, of Baltimore, recently read a paper and gave a clinic before the Pennsylvania Odontological Society, demonstrating a new method of constructing crowns. We call especial attention to this, as the method is an exceedingly simple one, and is capable of easy modification to meet the exigencies which occur in practice.

---

Oral Hygiene.—In the March issue of the *Dental Cosmos*, Dr. Kirk contributes an excellent editorial on the subject of "The Hygiene of the Mouth." It is such plain, practical papers that are the most helpful to the average practitioner and student. The doctor urges renewed efforts from the profession in the way of researches upon the subject, and among other things says, "The value of a careful and accurate study of the best means of securing immunity from diseases, both general and local, by means of suitable antiseptic and hygienic measures, with reference to the oral cavity, is unquestionable. That the best means at present in popular vogue is insufficient will not be gainsaid. More light is needed in this direction. Shall it not come from our own ranks?"

